# Application of Novel Thermal Technology in Foods Processing

**DOI:** 10.3390/foods11010125

**Published:** 2022-01-05

**Authors:** Sze Ying Leong, Indrawati Oey

**Affiliations:** 1 Department of Food Science, University of Otago, Dunedin 9054, New Zealand; sze.leong@otago.ac.nz; 2 Riddet Institute, Palmerston North 4442, New Zealand

Advanced and novel thermal technologies, such as ohmic heating and dielectric heating (e.g., microwave heating and radio frequency heating), have been developed to improve the effectiveness of heat processing whilst warranting food safety and eliminating undesirable impacts on the organoleptic and nutritional properties of foods. Novel thermal technologies rely on heat generation directly inside foods, which has implications for improving the overall energy efficiency of the heating process itself. The use of novel thermal technologies is dependent on the complexity and inherent properties of the food materials of interest (e.g., thermal conductivity, electrical resistance, water content, pH, rheological properties, food porosity, and presence of particulates). Moreover, there is a need to address the combined use of thermal processing with emerging technologies such as pulsed electric fields, high hydrostatic pressure and ultrasound to complement the conventional thermal processing of fluid or solid foods.

This Special Issue provides readers with an overview of the latest applications of various novel technologies in food processing. A total of eight cutting-edge original research papers and one comprehensive review paper discussing novel processing technologies from the perspectives of food safety, sustainability, process engineering, (bio)chemical changes, health, nutrition, sensory issues, and consumers are covered in this Special Issue.

Drying is a conventional thermal processing technique that is very effective in prolonging the shelf life of a food product by reducing microbial activities while facilitating its transportation and storage by decreasing the product weight and volume. The long drying time and decline in the product quality with drying duration has driven an urgent need to resolve these issues. Two approaches have recently been proposed: (i) the application of pre-treatments such as microwave (MW) or ultrasound (US) on raw material prior to drying [1], and (ii) the development of either a hybrid drying process involving convective-infrared (IF) [1] or dielectric drying involving MW and radio frequency (RF) [2]. Using turnip slices as a case study, the work of Taghinezhad et al. [1] explored several independent variables such as pretreatments applied to the raw material prior to drying (MW [360 W for 2.5 min], US [30 °C for 10 min] and blanching [90 °C for 2 min], the temperature of the drying air (50, 60, and 70 °C) and the thickness of the materials (2, 4, and 6 mm) on the response variables including the quality indices (color difference and shrinkage) and drying factors (drying time, effective moisture diffusivity coefficient, specific energy consumption (SEC), energy efficiency and dryer efficiency) using a hybrid convective-IF dryer. The response surface method was used to optimize the drying process and the response variables were predicted by the adaptive neuro-fuzzy inference system model. The results indicated that an increase in the dryer temperature and a decline in the thickness of turnip slices can enhance the evaporation rate, which will decrease the drying time (from 40 to 20 min), SEC (from 168.98 to 21.57 MJ/kg), color difference (from 50.59 to 15.38) and shrinkage (from 67.84% to 24.28%) while increasing the effective moisture diffusivity coefficient (from 1.007 to 8.11 × 10^−9^ m^2^/s), energy efficiency (from 0.89% to 15.23%) and dryer efficiency (from 2.11% to 21.2%). Compared to US and blanching, MW pretreatment increased the energy and drying efficiency, while the variations in the color and shrinkage of products were the lowest in the US pretreatment. In another drying example using pecan nut kernels, the work of Zhang et al. [2] considered the dielectric properties (DPs) of raw material, an important factor in the design of effective MW and RF drying processes. The DPs of raw materials were investigated over frequencies ranging from 10 to 3000 MHz at moisture contents between 10 to 30% in a temperature range of 5–65 °C at varying salt strengths (mild, medium, and heavy). It was found that the dielectric constant (ε′) and loss factor (ε″) of the kernels decreased significantly with increasing frequency in the RF band, but decreased gradually in the measured MW band. The moisture content and temperature increase greatly contributed to the increase in the ε′ and ε″ of samples, and ε″ increased sharply with increasing salt strength. Quadratic polynomial models were established to simulate DPs as functions of temperature and moisture content at four frequencies (27, 40, 915, and 2450 MHz), with R^2^ > 0.94. The average penetration depth of electromagnetic energy into the pecan kernels in the RF band was greater than that in the MW band (238.17 vs. 15.23 cm).

MW pasteurization and sterilization is an emerging thermal technology that combines preheating with hot water and MW energy (915 MHz) to achieve sterilization within a shorter time. However, a well-reported challenge with the MW processing of food is non-uniform heating, which could lead to the formation of cold spots in the processed products and is consequently unable to achieve the targeted sterilisation efficiency. Two research works from Soni et al. [3,4] addressed these food safety issues using novel validation method and tools. In their first study [3], novel spore pouches were developed using mashed potato as a food model inoculated with either *Geobacillus stearothermophilus* or *Clostridium sporogenes* spores to evaluate the sterilization efficiency of Coaxially induced MW pasteurization and sterilization (CiMPAS). The spore pouches were placed at pre-determined specific locations, especially cold spots, in each food tray before being processed using two regimes (R-121 and R-65), which consisted of 121 °C and 65 °C at 12 and 22 kW, respectively, followed by recovery and enumeration of the surviving spores. To identify cold spots or the inoculation location, mashed potato was spiked with Maillard precursors and processed through CiMPAS, followed by measurement of lightness (L*) values. Inactivation equivalent to of 1–2 and >6 Log CFU/g for *G. stearothermophilus* and *C. sporogenes* spores, respectively, was obtained on the cold spots using R-121 (total processing time of 64.2 min). Inactivation of <1 and 2–3 Log CFU/g for *G. stearothermophilus* and *C. sporogenes* spores, respectively, on the cold spots was obtained using R-65 (total processing time of 68.3 min), whereas inactivation of 1–3 Log CFU/g of *C. sporogenes* spores was obtained on the sides of the tray. The results were reproducible across three processing replicates for each regime, and inactivation at the specific locations was clearly distinguishable. For their second study [4], hyperspectral imaging (HSI) was used to identify cold spots in CiMPAS-treated mashed potato and directly compared to the results of color changes due to the Maillard reaction after MW-induced sterilization. To visualize the HIS spectra of each tray in comparison with the control sample (raw mashed potato), the mean spectrum (i.e., mean of region of interest) of each tray, as well as the control sample, was extracted and then fed to the fitted principal component analysis model. The HIS results coincided with those post hoc analysis of the average reflectance values. Despite the presence of a visual difference in browning, the L* values were not significantly different to detect a cold spot among a range of 12 processed samples. At the same time, HSI could identify the colder trays among the 12 samples from one batch of microwave sterilization.

Apart from MW processing, the use of ohmic heating is another alternative technology to sterilize and pasteurize heat sensitive food products that can provide better thermal uniformity, a high heating rate and energy efficiency. The work of Joe et al. [5] took a novel approach developing an ohmic–vacuum combination (OH-VC) heating system to process a multiphase type of senior-friendly food. Changes in the physical and electrical properties of senior-friendly model foods were investigated depending on the experimental conditions such as vacuum pressure intensity and vacuum pretreatment time. Numerical simulations based on the experimental conditions were performed using COMSOL Multiphysics software. The OH-VC heating method with agitation reduced the heating time of the model food, and non-uniform temperature distribution in model food was successfully resolved due to the effects of vacuum and agitation. Furthermore, difference was found in the hardness of solid particles depending on the vacuum treatment time and intensity after the heating treatment.

In recent years, the use of pulsed electric field (PEF) technology has gained in popularity, particularly in the potato industry for the production of French fries and potato crisps, to “condition” the raw material (potato tubers) prior to subsequent processing (i.e., cutting, blanching and frying). However, the influence of PEF pretreatment on the frying process and related chemical reactions for food materials is still not fully understood. PEF treatment of plant tissue causes structural modifications, which are likely to influence heat, mass and momentum transfers, as well as alter the rate of chemical reactions during the frying process. Detailed insights into the frying process in terms of heat, mass (water and oil) and momentum transfers are outlined in a comprehensive review article by Xu et al. [6], in conjunction with the development of the Maillard reaction and starch gelatinization during frying. These changes occur during frying, and consequently impact oil uptake, moisture content, colour, texture and the amount of contaminants generated in fried foods, as well as the fried oil. Therefore, the effects of PEF pretreatment on these properties across a variety of fried plant-based foods are summarised in the review article. The different mathematical models used to potentially describe the influence of PEF on the frying process of plant-based foods and predict the quality parameters of fried foods produced from PEF-treated plant materials are also addressed in the review article.

In agreement with the review article by Xu et al. [6], the work of Gholamibozanjani et al. [7] conducted a timely investigation on the use of suitable heat and mass transfer models to predict temperature distribution during potato frying after pre-treatment with PEF. Meanwhile, the work of Abduh et al. [8] reported the kinetics of colour development during the frying of four potato cultivars pre-treated with PEF, in which the kinetic result can later aid in the optimisation of frying conditions for deep-fried potato industries where PEF technology is implemented. Based on an unsteady-state heat conduction, a mathematical model was developed by Gholamibozanjani et al. [7] to describe the simultaneous heat and moisture transfer during potato frying. For the first time, the equation was solved using both enthalpy and Variable Space Network methods, based on a moving interface defined by the boiling temperature of water in a potato disc during frying. Two separate regions of the potato disc, namely fried (crust) and unfried (core), were considered heat transfer domains. A variable boiling temperature of the water in potato discs was required as an input parameter for the model. This is because water is continuously evaporated during frying, resulting in an increase in the soluble solid concentration of the potato sample. The application of PEF pretreatment prior to frying had no significant effect on the measured moisture content, thermal conductivity or frying time compared to potatoes that did not receive PEF pretreatment. However, a PEF pretreatment at 1.1 kV/cm and 56 kJ/kg reduced the temperature variation in the experimentally measured potato center by up to 30%. On the other hand, the effect of PEF (1 kV/cm; 50 and 150 kJ/kg) followed by blanching (3 min., 100 °C) on the colour development (L*) of potato slices during frying was studied on a kinetic basis by Abduh et al. [8]. Four potato cultivars, ‘Crop77’, ‘Moonlight’, ‘Nadine’, and ‘Russet Burbank’, with different glucose and amino acid contents, were used. The implementation of PEF and blanching as a sequential pre-treatment prior to frying was found to be effective in improving the lightness of the fried products for all potato cultivars. PEF pre-treatment did not change the kinetics of L* reduction during frying (150–190 °C), which followed first-order reaction kinetics. The estimated reaction rate constant (*k*) and activation energy (*E_a_* based on Arrhenius equation) for non-PEF and PEF-treated samples were cultivar-dependent. The estimated *E_a_* values during the frying of PEF-treated ‘Russet Burbank’ and ‘Crop77’ were significantly lower (up to 30%) than their non-PEF counterparts, indicating that the change in the *k* value of L* became less temperature-dependent during frying. 

The use of PEF can be also extended to red winemaking to achieve a similar outcome as the pre-fermentation heating (or thermovinification) of red grapes, which is a common practice in commercial wineries in Europe. PEF treatment applied to red grapes before the maceration-fermentation stage allows for a reduction in the contact time of grape skins, and helps to obtain wines with a higher polyphenolic content without involving heat in the vinification process. The work of Maza et al. [9] monitored the evolution of the polyphenolic compounds and sensory properties of wines obtained from Grenache grapes, either untreated or treated with PEF, during the course of either bottle aging or oak aging followed by bottle aging. Immediately prior to aging in bottles or in barrels, enological parameters that depend on phenolic extraction during skin maceration were higher when grapes were treated with PEF. In terms of color intensity, phenolic families, and individual phenols, the wine obtained with grapes treated by PEF followed a similar evolution to untreated control wine in the course of aging. Sensory analysis revealed that the application of a PEF treatment resulted in wines that are sensorially different, where panelists preferred wines obtained from grapes treated with PEF. Physicochemical and sensory analyses showed that grapes treated with PEF are suitable for obtaining wines that require aging in bottles or in oak barrels. 

In summary, all the papers published in this Special Issue highlighted a large portion of the research activities in the field of advanced thermal processing, aiming to improve the efficiency of food processing, ensure food safety, enhance product quality and reduce food waste. The development of novel thermal-processing technologies and an exploration of the combined use of nonthermal processing to complement thermal processing will remain a very active research area in the coming decades. Future studies should consider the feasibility and applicability of these technologies or processing approaches for industrial-scale environments.
